# Forensic Biochemical Markers to Evaluate the Agonal Period: A Literature Review

**DOI:** 10.3390/molecules26113259

**Published:** 2021-05-28

**Authors:** Enrica Rosato, Martina Bonelli, Marcello Locatelli, Ugo de Grazia, Angela Tartaglia, Fabio Savini, Cristian D’Ovidio

**Affiliations:** 1 Department of Pharmacy, University of Chieti-Pescara “G. d’Annunzio”, Via dei Vestini 31, 66100 Chieti, Italy; enrica.rosato@unich.it (E.R.); marcello.locatelli@unich.it (M.L.); angela.tartaglia@unich.it (A.T.); 2 Section of Legal Medicine, Center for Advanced Studies and Technology (CAST), Department of Medicine and Aging Sciences, University “G. d’Annunzio” of Chieti-Pescara, Via Polacchi, 66100 Chieti, Italy; martina.bonelli@unich.it; 3 Laboratory of Neurological Biochemistry and Neuropharmacology, IRCCS Neurological Institute Foundation Carlo Besta, Via Celoria 11, 20133 Milan, Italy; ugo.degrazia@istituto-besta.it; 4 Pharmatoxicology Laboratory-Hospital “Santo Spirito”, Via Fonte Romana 8, 65124 Pescara, Italy; fabio.savini@ausl.pe.it

**Keywords:** post-mortem analysis, thanatobiochemistry, agonal period, post-mortem interval, biochemical markers

## Abstract

Currently, forensic research is multidisciplinary with new methods and parameters useful to define the cause and time of death as well as survival/agony times. The identification of biochemical markers able to estimate agonal period has been studied by many forensic researchers. It is known that the estimation of agonal time in different types of death is not always easy, hence our interest in literature’s data. The studies analyzed in this review confirm the important role of thanatobiochemistry for the estimation of survival times. Regardless of the death cause, the survival/agony time between the primary event and death influences markers concentrations in biological samples (e.g., blood, urine, cerebrospinal fluid). Different biomarkers can be used for qualitative evaluations in deaths with short and long agony (e.g., C-reactive protein, ferritin, GFAP, etc.). Instead, the quantitative interpretation showed limits due to the lack of reference cut-offs. Thanatobiochemistry is a useful tool to confirm what emerged from autopsies findings (macroscopic and histological analysis), but further studies are desirable to confirm the evidence emerging from our review of the literature.

## 1. Introduction

The determination of the death cause and post-mortem interval (PMI) are the main aspects of interest in forensic research by using new objectives and no biased parameters and indicators [1,2].

The technical applications in forensic medicine are very widespread; it can be used histological, toxicological, biochemical techniques, analytical and bioanalytical procedures, or more recently, molecular biology, metabolomics, proteomic and transcriptomic techniques to analyze tissues and biological materials samples (e.g., blood, urine, cerebrospinal fluid, vitreous humor, etc.) collected during autopsies.

In this context, the possibility to detect one or more parameters able to estimate the time of agony/survival between the primary event (e.g., traumatic event) and death has a primary role in understanding the agonal mechanism and the PMI.

The vital functions (respiratory and cardiocirculatory) are compromised during the terminal phase before the death. Theoretically, the Hardy scale [3] classify the agonic period in:

(1) Fast and violent death (e.g., accident, blunt force trauma or suicide) with terminal phase estimated in less than 10 min.
(2) Fast death of natural causes with terminal phase estimated in less than 1 h (but more than 10 min).
(3) Intermediate death with a terminal phase ranging from 1 to 24 h.
(4) Slow death with a terminal phase longer than 24 h.

In forensic practice, it is important to estimate the agonal period, even if it is difficult due to the scarce circumstantial and pathological evidence during the external cadaveric inspection and/or autopsy.

The overall data emerging from the research on biochemical, histological, histochemical and immunohistochemical markers capable of defining the time interval between the onset of a hypoxic-ischemic insult and death seem fragmentary without a shared opinion on this topic [4].

Post-mortem analysis of biochemical markers could be useful to define death causes and to estimate the agonal period even if biochemical methods for this forensic purpose have several limitations. 

The interpretation of biochemical post-mortem results could be affected by interference due to pre-existing factors disorders during life, cause of death, survival period, post-mortem changes (e.g., cell deterioration), environmental factors, chemical properties, distributions and locations of the analytes, and on analytical procedures [5,6].

The analysis of samples collected during autopsies shows qualitative and quantitative limits related to the inevitable post-mortem phenomena such as hemolysis, acidification, proteolysis and post-mortem redistribution processes that could modify the results. Another limitation derived from the absence of a quantitative reference range, so the comparison between post-mortem results with the same marker value on living is very difficult [7]. However, several studies have positively suggested the possibility of using multiple markers for forensic purposes by setting a post-mortem reference range considering agonal and post-mortem changes [8].

Hence, the need to identify post-mortem reference values and markers concentration limits useful for forensic investigations in order to provide a valid interpretation of the results obtained [9].

By focusing on the thanatochemistry published studies and based on the actual knowledge, this review shows for the first time a literature overview on available biochemical markers for the agonal period estimation. 

## 2. Materials and Methods

Specifically, this narrative review was performed using the PUBMED database with search criteria such as “agonal time, biochemistry for agonal time, biochemical markers to estimate the time of agony.”

We also examined the bibliography of the selected articles to identify further studies of interest. We considered only articles published in English; if only the abstract was available, we excluded it.

The selected articles are related only to biochemical applications, while histological and/or immunohistochemical tissue characterization studies were excluded. In this overview, studies on guinea pigs and on cadavers without distinction by death type, biological samples (blood, urine, cerebrospinal fluid-CSF, etc.) and analytical technique (enzyme-linked immunosorbent assay-ELISA, enzymatic assay, HPLC) were included.

## 3. Forensic Biochemical Markers to Evaluate Agonal Period 

### 3.1. C-Reactive Protein and Acute Phase Markers

The C-reactive protein (CRP) is a clinical marker of immune response activation. This protein is produced and secreted in response to the activation of the acute phase reaction, with an increase in its circulating levels from trace (0.1 µg/mL) to several hundred µg/mL. 

This feature makes it a very useful clinical marker in the diagnosis of infection and tissue inflammation [10].

It also seems to be applicable in forensic investigations for postmortem diagnostic purposes and to estimate survival times in order to differentiate between acute and non-acute death. 

This protein is a useful postmortem marker with increased and detectable values in many pathological conditions such as sepsis, acute pancreatitis, pneumonia, pulmonary embolism, traumatic injury, acute myocardial infarction and death due to alcoholic ketoacidosis in chronic abusers [11,12].

Thanks to its stability without serum post-mortem modifications, CRP value seems to be important for the evaluation of the agonal period [13].

Masaki et al. [14] have studied CRP post-mortem serum levels in different types of death (blunt trauma, stab and gunshot wounds, asphyxia, drowning, intoxication, burning, hypothermia, hyperthermia, electrocution and natural death) with different survival/agony times (acute death within 30 min and non-acute death more than 30 min). They observed that the increase of CRP post-mortem levels was related to the survival time and to the septic factors. CRP serum levels were increased in non-acute deaths (192 cases of non-acute death; median concentration of 3.68 mg/dL) while remaining low in acute deaths (216 cases of acute death; median concentration of 0.12 mg/dL).

This trend was also reproducible by stratifying the acute deaths in immediate and non-immediate so that in all immediate deaths (15 cases out of 16) and in 13.5% of non-immediate deaths, the CRP did not showed elevation with values below 0.5 mg/dL while 23% of the non-immediate deaths had elevation with CRP levels up to 0.5 mg/dL.

A confounding factor for the use of CRP as a marker of survival times could derive from the physiological pattern of the CRP release into circulation after traumatic insult (levels increase 6–12 h after the “external” traumatic or infectious insult), so if death occurs in the initial phase after the insult, an increase in the CRP cannot be expected [15].

However, other studies suggest that a low post-mortem level of CRP could be a detector of deaths with short agony.

Astrup et al. [11], with studies on different types of death (including blunt trauma, myocardial infarction, ante-mortem surgery), observed that CRP post-mortem levels were normal in a short survival time (death within 1 h), while in a prolonged survival period (death ranging from 1 to 22 days), the levels increased even when the cause was not septic.

Ondruschka B. et al. [13] have studied post-traumatic death (head trauma) and confirmed serum (and cerebrospinal fluid) CRP function. They classified the survival time in acute (death within 2 h), sub-acute (death ranging from 2 h to 3 days) and chronic (death after 3 or more days) and observed a linear correlation between CRP levels and survival time with an increased concentration in deaths with longer survival periods (CRP post-mortem serum range from 0 to 1.2 mg/dL).

The overall results suggest that the CRP post-mortem increase could be interpreted as a vital reaction in deaths with a long survival period regardless of the type of death.

On the other hand, the quantitative interpretation is less reliable because the reference range is not always reproducible between the different studies, and this is not surprising considering that in post-mortem analyses, the cut-offs and ante-mortem samples were not specified for comparison.

Other biochemical markers related to the acute phase showing a tendency to increase both in serum and CSF samples in traumatic head deaths with a long survival time.

For example, serum levels of soluble tumor necrosis factor receptor type 1 (sTNFR1) do not increase in deaths with a survival time of less than 3–4 days (values range of 0–25 ng/mL) [13]. Additionally, Interleukin-6 (IL-6) has shown a correlation with survival time. In deaths with a long survival period, IL-6 was found low values in serum and CSF, so if its degradation occurs in a short time, its presence in post-mortem samples could be related to a short agonal period.

### 3.2. Ferritin

Ferritin is a protein involved in iron storage. This marker is useful for clinical purposes in the evaluation of many diseases such as iron deficiency anemia and iron-overload conditions, clinical inflammatory conditions and trauma [16].

Ferritin has also been studied as a possible post-mortem biomarker useful in estimating the agonal period after traumatic head injury.

Ondruschka B. et al. [13] observed that CSF ferritin concentration is influenced by survival time after brain injury, and in particular, the biomarker was only detected in deaths with survival time of at least 2 h (levels greater than 30.0 mg/L), so in the case of short agony, ferritin is not detected. The same consideration was also made for serum levels. 

Despite these findings, ferritin post-mortem value must be interpreted with caution. Before confirming ferritin post-mortem use, further studies are necessary to evaluate the existence of inter-individual differences with the increase of post-mortem interval [17] and if this marker has the same behavior in other types of death.

### 3.3. Thyroglobulin and Thyroid Hormones

The thyroid hormones, triiodothyronine (T3) and thyroxine (T4), are tyrosine-based hormones produced by the thyroid gland that are primarily responsible for normal development, growth, cellular differentiation and metabolic regulation. Excess or deficiency of thyroid hormones (hyper- and hypo-thyroidism) has important effects on these functions [18]. Clinically, their dosage is indicated for the evaluation of thyroid function in all thyroid diseases.

Thyroglobulin (TG) is a specific protein produced by thyroid follicular cells. Generally, this protein is stored in the follicular lumen, and under the action of thyroid-stimulating hormone (TSH), it is reabsorbed by the follicular cells and hydrolyzed with thyroid hormone release. In physiological conditions, TG is present in serum in small quantities; its dosage is not useful for evaluating thyroid function. 

Many studies have been conducted on thyroid biomarkers for the forensic diagnostic purpose (often in asphyxia deaths) and for PMI estimation. Some authors have evaluated these markers for agonal period definition in different types of death.

Muller et al. [19] have compared cases of violent mechanical asphyxia and instant post-traumatic deaths due to aortic arch rupture or heart injury.

The analyses on cardiac blood samples showed the highest serum concentrations of thyroglobulin in deaths from strangulation (mean value of 561.6 ng/mL) while the lowest in immediate post-traumatic deaths without agony (mean values of 23.3 ng/mL).

This suggested that the post-mortem serum levels of thyroglobulin could be related to the agonal period, and its serum increase was indicative of a residual vital process due to prolonged time of agony.

Tani et al. [20] studied different types of deaths and revealed that asphyxia and traumatic acute/sub-acute brain injury were associated with increased serum levels of free triiodothyronine (fT3), free thyroxine (fT4) and thyroglobulin.

An aspect of interest was observed for deaths due to cardiogenic and hemorrhagic shock as possible causes of secondary cerebral hypoxia/ischemia without serum fT3 and fT4 increase. The explanation could depend on the different survival periods. Therefore, in deaths with a short agony, the thyroid hormones increase was not observed due to the reduced period of exposure of the brain to hypoxia/ischemia. However, the post-mortem use of thyroid markers in the estimation of agony times requires particular attention in the collection phase because the analytical results can be influenced by the sample type (e.g., cardiac blood vs peripheral blood), and in the interpretation of the results, thyroglobulin and thyroid hormone levels may be affected by post-mortem changes (e.g., autolysis) [21].

### 3.4. Serum Protein S100B and Neuronal Specific Enolase (NSE)

The S100B protein belongs to a family of calcium-binding proteins and is generally found in brain tissue for 80–90% of its pool (mainly in astrocytes, oligodendrocytes and Schwann cells of the peripheral nervous system), while the remainder is expressed in non-neuronal tissues such as adipose tissue, cartilage and skin.

This protein is involved in different neuropathological conditions such as acute brain injury, neurodegenerative and psychiatric diseases (e.g., Alzheimer’s disease, schizophrenia) or oncological diseases (e.g., astrocytoma, schwannomas or melanomas).

Clinically, serum S100B increases from brain damage and other pathological conditions such as septic and hemorrhagic shock, cardiac arrest, cardiac surgery and others. 

The neuron-specific enolase (NSE) is a specific form of the glycolytic enzyme enolase. It is a soluble cytoplasmic protein localized in intra- and extra-cranial neurons and peripheral neuroendocrine cells, and it is present in platelets and erythrocytes.

Serum NSE is a marker of brain damage with an increase after trauma, but its increase is observed also after hemorrhagic shock and ischemia [22].

The serum protein S100B and neuronal specific enolase (NSE) have been studied for forensic purposes in deaths related to traumatic brain injury (TBI) to determine the severity of brain damage and the survival time after fatal trauma. These markers have shown the advantage of being stable in cerebrospinal fluid at least within the first 5 days after death.

In serum, only the S100B protein shows stability for at least 48 h after death [23], and for this reason, this marker analysis is recommended for this interval to avoid passive increase due to the post-mortem effects of cell destruction and proteolysis [22]. The NSE analysis in serum could give drawbacks in results interpretation because this biomarker is also found in erythrocytes. In this last case, post-mortem hemolysis could produce false information.

Ri et al. [23] observed that in head trauma, the serum levels of S100B were influenced by the brain injury severity and survival time, so it became significantly higher in deaths with the survival of less than 6 h compared to the longest survival times. The reduction in serum S100B levels in cases of longer survival was mainly attributed to its short half-life (3–4 h). In cerebrospinal fluid, S100B levels did not show a correlation with survival times even if a post-mortem increase within 48 h was observed for acute cardiac death and asphyxia [24]. 

Out of a total of 40 autopsies with various causes of death, Ondruschka et al. [22] found CSF S100B levels above 10,000 ng/mL after fatal TBI with a survival time of at least 20 min, while CSF NSE levels above 6000 ng/mL after fatal TBI with survival times ranging from 15 min to 5 days.

Sieber et al. [25] also observed that NSE and S100B levels in CSF were elevated in deaths related to TBI, while serum levels did not show significant differences in the types of death analyzed together with TBI (e.g., myocardial infarction, diffuse cerebral hypoxia, traumatic death without head injury). This suggests the contribution of other factors such as cerebral hypoxia and/or the passive release of proteins during the agony phase or in the early post-mortem period together with the direct traumatic effect on nervous tissue. In fact, in traumatic head deaths, the levels of CSF markers increase with the longest survival times, reaching the maximum level within the first 3 days after the trauma (sub-acute death with survival time 2 h–3 days) with a survival time of at least 30 min. The same marker has a similar trend in the serum. This could indirectly indicate that in immediate deaths, regardless of the causes, these markers have the lowest levels.

Although NSE and S100B serum assay is not recommended for thanatodiagnostic purposes, it could become a useful tool in estimating survival times.

### 3.5. Glial Fibrillary Acidic Protein (GFAP) 

The glial fibrillary acidic protein (GFAP) is an intermediated filament protein produced by astrocytes with expression in brain tissue, responsible for cell stability and shape [26].

In normal conditions, this protein is not detectable in the circulation, but after significant damage to the central nervous system (e.g., astroglial and cellular necrosis), it can be released into the cerebrospinal fluid and therefore into the blood. In particular, GFAP serum elevation is observed after intracerebral hemorrhage and traumatic brain injury or ischemic and hypoxic injury. 

Generally, serum GFAP levels correlate with the gravity of brain tissue destruction, and it is useful in forensic practice for agonal period estimation thanks to its high post-mortem stability.

Breitling et al. [27], in their study, observed the absence of differences in GFAP levels between brain death (median value 0.96 μg/L) and other causes (median value 1.21 μg/L). At the same time, it was observed that the increase in survival time (from a few minutes to hours) was associated with higher serum GFAP concentration (median value 1.76 μg/L) compared to deaths with short survival (from a few seconds to minutes; median value 0.58 μg/L) and immediate (a few seconds; median value 0.21 μg/L).

GFAP blood release suggested a vital reaction due to the protein flow from the damaged nervous tissue to the cerebrospinal fluid and then into the blood through the blood-brain barrier, consequently, in sudden death, the immediate cessation of vital functions is mainly responsible for the lowest serum levels found. Although the marker is specific for nervous tissue, its finding in the serum is not specific for traumatic head death, and it can be used for any type of disease, opening an interesting feature in forensic practice. In addition, for this biomarker, no cut-offs for the interpretation of results were reported. In this case, the difficulty is greater and related to the absence of reference range from livings.

### 3.6. Liver-Type Fatty Acid-Binding Protein (L-FABP) and 8-Hydroxy-2-Deoxyguanosine (8-OHdG) 

Liver-type fatty acid-binding protein (L-FABP) is a protein normally expressed in renal proximal tubule cells and is partly excreted in the urine. Urinary L-FABP is a clinical marker useful in chronic renal disease for monitoring and predicting the progression of renal disease (e.g., chronic glomerular disease) and in acute kidney injury such as a renal tubular injury due to septic shock with levels significantly elevated [28,29].

8-hydroxy-2-deoxyguanosine (8-OHdG) is another biomarker that derives from the oxidization of DNA (deoxyguanosine) by free radicals; it is excreted in the urine. Scientific evidence has highlighted that this parameter is very useful for clinical purposes because it reflects cellular oxidative damage produced by stress factors. For example, an elevated level of urinary 8-OHdG is observed in patients with cancer, atherosclerosis and diabetes. Moreover, it also shows a correlation with the severity of chronic complications (e.g., nephropathy and retinopathy) [30].

A study [31] highlighted the forensic use of urinary detection of L-FABP and 8-OHdG as potential additional biomarkers for agony time estimation. These markers are not influenced by post-mortem interval and are more stable in the urine than in the blood. 

These biomarkers, corrected for urinary creatinine, showed a tendency for L-FABP/Cr to maintain low levels in case of suspected short agony with death within 1 h, while 8-OHdG/Cr can increase when the agony period is longer (death beyond 24 h). Therefore, the L-FABP level below the cut-off could indicate a shorter agonal period, while the 8-OHdG above the cut-off could indicate a longer agonal period. Starting from these results, further studies are desirable. In Table 1 were reported the proposed cut-off values for these markers.

### 3.7. Catecholamines

The catecholamines (dopamine, norepinephrine and epinephrine) are a class of chemical neurotransmitters and hormones with an important function in the regulation of physiological processes and in the development of neurological, psychiatric, endocrine and cardiovascular diseases.

Adrenaline and noradrenaline are mainly produced by adrenal glands in response to a stress condition, and instead, dopamine is a central nervous system neurotransmitter produced by neurons of substantia nigra. 

Adrenaline prevails in the adrenal medulla; noradrenaline prevails in the peripheral nervous system and in SNC with dopamine. 

An increase in their blood levels is associated with various diseases that involve neurodegeneration of central and peripheral, catecholamine neuronal systems, also with traumatic brain injury, stress factors and catecholamine-producing tumors [32].

**Figure 1:**
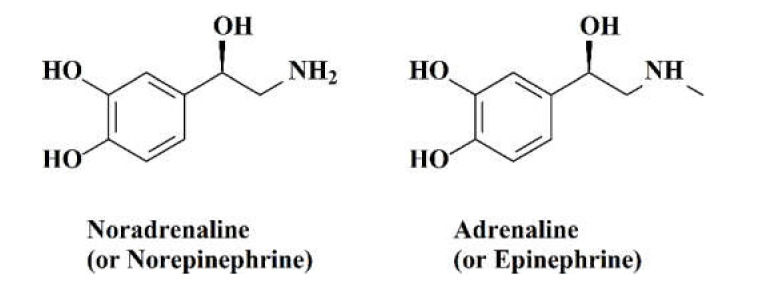
Chemical structures for the evaluated catecholamines.

Serum and urinary catecholamines (Figure 1) are well-studied biomarkers also for forensic purposes. The results that emerged for the estimation of agonal time are in contrast. Some results suggest that the increase in serum catecholamines levels is useful for estimating the agonal period, and in particular, norepinephrine and epinephrine are increased in deaths with short agony comparing to deaths with a long agonal period (norepinephrine 5.4 ± 2.6 ng/mL vs. 2.8 ± 0.1 ng/mL; epinephrine 6.0 ± 3.4 ng/mL vs. 3.8 ± 3.0 ng/mL) [17].

Other studies suggest that the absolute blood value of epinephrine and norepinephrine (cardiac and peripheral blood) does not show significant differences related to the cause of death and to the agonal period [33].

Therefore, today post-mortem analysis of catecholamines in various biological samples (blood, urine, cerebrospinal fluid) is not used either for thanatodiagnostic purposes or to estimate agonal time regardless of death causes [34,35]. The main confounding factor in the use of the serum dosage of catecholamines is the poor stability related to post-mortem autolysis phenomena. Catecholamines have been extensively studied in deaths from hypothermia and, for this reason, their possible forensic application is to provide information on death with exposure to low temperatures [36]. 

### 3.8. Analytical Methods for Biochemical Markers Evaluation

The quality of cadaveric samples can be altered due to post-mortem contamination and decomposition phenomena such as autolysis or putrefaction. Despite these limits, many analytical techniques generally used for clinical diagnostic purposes can be used also for forensic analysis with good results. In Table 2, the most recently applied procedures for biochemical markers evaluation are reported with their main characteristics.

## 4. Conclusions

This review is focused on biochemical parameters useful for forensic purposes. Based on the emerged evidence, some of these biomarkers showed validity not only for diagnosis of death cause such as traumatic head injury and asphyxia but also for agonal period estimation. In fact, biomarkers in biological samples (blood, urine and cerebrospinal fluid) seem to be influenced by survival/agony time between the primary event and death.

In particular, in deaths with longer agony, the reported studies revealed that serum and cerebrospinal fluid levels of C-reactive protein are increased in death with a survival time of at least 30 min–1 h. 

In traumatic death due to head injury, we observed an increase of serum and cerebrospinal fluid levels of ferritin in a survival time of at least 2 h after a head injury, while serum and cerebrospinal fluid levels of sTNFR1 are increased in a survival time of at least 3–4 days.

Cerebrospinal fluid levels of S100B and NSE are increased, respectively, in death within 3 days, but with a survival time of at least 30 min and in death within 5 days, but with a survival time of at least 15 min after a head injury. 

For thyroid biomarkers, serum levels of thyroglobulin and thyroid hormones showed an increase regardless of death causes, despite no survival time references are indicated. Moreover, it is possible to observe an interesting role of urinary levels of 8-OHdG that showed a non-specific increase in death beyond 24 h.

In deaths with a short agony after a head injury, the studies reported an increase in serum and cerebrospinal fluid levels of IL-6 without specific survival time, an increase in serum levels of GFAP with survival from minutes to hours and an increase in serum levels of S100B with survival less than 6 h.

Moreover, it is possible to observe a decrease in urinary levels of L-FABP in death within 1 h regardless of the type of death.

The post-mortem quantitative interpretation of these biomarkers is more difficult due to the lack of cut-offs and ante-mortem data.

Despite the limitations, these results confirm the important role of thanatobiochemistry and analytical techniques applicable in Forensic Medicine, so further studies in this direction are desirable.

Starting from this evidence, the future direction is to focus the attention on ideal biochemical parameters. The ideal marker is not affected by analytical interferences due to post-mortal phenomena and the presence of unknown intrinsic factors at the time of death. For example, pre-existing pathologies could change the interpretation of the laboratory data (thyroid disease and post-mortem levels of thyroid hormones).

Moreover, the ideal marker for the estimation of agony should be reproducible for all types of death. 

In this direction, it is interesting to increase the studies on the biomarkers that seem to show these characteristics, such as urinary L-FABP and 8-OHdG.

The study of these markers and others is desirable in order to obtain new objective laboratory parameters useful for forensic and justice purposes.

## Figures and Tables

**Table 1:** Proposed cut-offs for the urinary biomarkers.

Biomarker	Matrix	Cut-Off	Agonal Period
L-FABP/Cr	Urine	<31.3 µg/g Cr	within 1 h
8-OHdG/Cr		<17.8 µg/g Cr	within 24 h

**Table 2:** Recently applied procedures for biochemical markers evaluation.

Biochemical Marker	Methods	Matrix	Analytical Characteristics	**Ref.**
CRP	LC-MS/MS	Blood serum	LOD 67 pmol/L	[37]
Ferritin	LFIA	Blood serum	LOD 0.3 ng/mL Lowest visually detectable 0.05 ng/mL	[38]
	Electrochemical Immunosensor (ePAD)	Blood serum	LOD 0.19 ng/mL	[39]
	ICP-MS	Plasma	LOD 0.48 ng/mL	[40]
	ECLIA	Blood serum	LOD 0.5 ng/mL	[40]
T3 (fT3)	ECLIA	Blood serum	Minimum concentration detectability <0.30 nmol/L LOD 0.6 pmol/L; LOQ 1.5 pmol/L (error ≤ 30%)	[41]
			LOD 0.5 pmol/L; LOQ 3 pmol/L	[42]
	ELISA	Blood serum	Analytical sensitivity 37.4 pg/mL LOD N.R.; LOQ 2.9 pmol/L	[42]
	LC-MS/MS	Blood	LOD 0.4 pg/mL; LOQ 0.8 pg/mL	[43]
	LR-LC-MS	Blood	LOD 0.046 pmol/L; LOQ 0.154 pmol/L	[42]
	HR-LC-MS	Blood	LOD 0.0015 pmol/L; LOQ 0.0061 pmol/L	[42]
T4 (fT4)	ECLIA	Blood serum	Minimum concentration detectability <0.30 pmol/L LOD 0.5 pmol/L OQ 3 pmol/L (error ≤ 30%)	[41]
	ELISA	Blood serum	Analitycal sensitivity 0.29 ng/mL	[42]
	LC-MS/MS	Blood	LOD 0.025 ng/dL; LOQ 0.05 ng/dL	[43]
	HF-LPME/LC-MS	Blood serum	LOD 0.3 ng/g; LOQ 1 ng/g	[44]
	LR-LC-MS	Blood	LOD 1.8 pmol/L; LOQ 5.5 pmol/L	[42]
	HR-LC-MS	Blood	LOD 0.001 pmol/L; LOQ 0.0064 pmol/L	[42]
Thyroglobulin	ECLIA	Blood	N.R.	[20,21]
	ECLMA	Blood serum	Analitycal sensitivity 0.1 ng/mL	[45]
S100B	PEC immunosensor	Blood serum	LOD 0.15 pg/mL	[46]
	Electrochemical	Blood serum	AuEs LOD 18 pg/mL AuIEDs LOD 6 pg/mL	[47]
	Electrochemical	Blood serum	LOD 0.4 ng/mL	[48]
	CLIA	Blood serum	LOD 0.02 ng/mL	[49]
NSE	Electrochemical	Blood serum	LOD 0.6 ng/mL	[48]
GFAP	ELISA	Plasma (mice)	Lower LOD 9.0 pg/mL Lower LOQ 24.8 pg/mL Upper LOQ 16,533.9 pg/mL	[50]
	CLAISA	Blood serum	LOD 25 pg/mL	[51]
	Electrochemical	Blood serum	LOD 0.04 µg/mL	[52]
L-FABP	ELISA	Urine, Blood serum	N.R.	[31]
8-OHdG	MPA-FIA	Urine, blood serum	LOD 0.0008 µM	[53]
	LFIA	Urine	LOD 0.05 nM	[54]
Catecholamines	HPLC	Urine, blood serum	N.R.	[36]
	Electrochemical	Blood serum	NE LOD 196 nM; NE LOQ 312 nM	[55]
	Fluorescence	Blood serum	NE LOD 2.1 nM; EP LOD 6.8 nM	[56]
	LC-MS/MS	Plasma	EP, NE LOD 0.500 pg/mL EP Lower LOQ 5.00 pg/mL; NE Lower LOQ 20.0 pg/mL	[57]

*ECLIA:* Electrochemiluminescent immunoassay; *LFIA:* Lateral Flow Immunoassay; *ePAD:* Electrochemical paper-based analytical device; *ICP-MS:* Inductively coupled plasma-mass spectrometry; *ELISA:* Enzyme-linked immunosorbent assay; *LC-MS/MS:* liquid-chromatography-mass spectrometry; *LR-LC-MS:* low-resolution liquid chromatography-mass spectrometry; *HR-LC-MS:* high resolution liquid chromatography-mass spectrometry; *HF-LPME/LC-MS:* hollow fiber liquid-phase microextraction coupled with liquid chromatography-mass spectrometry; *ECLMA:* Electrochemiluminometric assay; *PEC:* photoelectrochemical immunoassay; *AuEs:* gold electrodes; *AuIEDs:* gold interdigitated electrodes; *CLIA:* chemiluminescence immunoassay; *CLAISA:* CD-linked antibody immunosorbent assay; *MPA-FIA:* multiple-pulse amperometric detection in flow injection analysis; *PC:* phosphocholines; *PE:* phosphoethanolamines; *NE:* norepinephrine; *EP:* epinephrine; *N.R.:* not reported.

## Data Availability

no data are linked to this review paper.

## Sample Availability

This paper is a review and no samples were involved in the study.
